# Strategic options for syphilis control in Papua New Guinea– impact and cost-effectiveness projections using the *syphilis interventions towards elimination (SITE)* model

**DOI:** 10.1016/j.idm.2021.03.004

**Published:** 2021-03-20

**Authors:** Shepherd Machekera, Peniel Boas, Poruan Temu, Zimmbodilion Mosende, Namarola Lote, Angela Kelly-Hanku, S. Guy Mahiane, Robert Glaubius, Jane Rowley, Anup Gurung, Eline Korenromp

**Affiliations:** a Government of Papua New Guinea, National Department of Health, AOPI Center, Waigani Drive, PO Box, 5896, Port Moresby, Papua New Guinea; b World Vision International, Ruta Place, Morata St, Gordons. P.O Box 4254, Boroko, National Capital District, Port Moresby, Papua New Guinea; c UNAIDS Papua New Guinea Country Office, Port Moresby, Papua New Guinea; d WHO Papua New Guinea Country Office, Communicable Disease & Health Emergency Dept., AOPI Centre, Waigani Drive, Port Moresby, Papua New Guinea; e Papua New Guinea Institute of Medical Research, 441 Homate Street, PO Box 60, Goroka, Eastern Highland Province, Papua New Guinea; f Kirby Institute, UNSW Sydney, Wallace Wurth Building, High Street, UNSW Australia Kensington, NSW 2052, Sydney, Australia; g Avenir Health, Modelling, Planning and Policy Analysis Dept., 655 Winding Brook Drive, Glastonbury, CT, 06033, USA; h Independent Consultant, 135 Gloucester Terrace, W2 6DX, London, UK; i Avenir Health, Modelling, Planning and Policy Analysis Dept., 150 Route de Ferney, PO box 2100, CH-1211 Geneva 2, Switzerland

**Keywords:** Syphilis, Prevention, Treatment, Cost-effectiveness, Resource allocation, National program strategy, ANC, antenatal care, FSW, Female Sex Worker, GUD, Genital Ulcer Disease, (I)BBS, (Integrated) Bio-Behavioural Survey, DHS, Demographic and Health Survey, MSM, Men who have sex with men, PNG, Papua New Guinea, PoM, Port Moresby, RPR, Rapid Plasma Reagin test, STI, sexually transmitted infection, TPHA, *Treponema pallidum* hemagglutination assay, TPPA, *Treponema pallidum* particle agglutination assay, VDRL, Venereal Disease Research Laboratory, WHO, World Health Organization

## Abstract

**Objectives:**

Papua New Guinea (PNG) has among the highest rates of sexually transmitted infections (STIs) globally and is committed to reducing their incidence. The Syphilis Interventions Towards Elimination (SITE) model was used to explore the expected impact and cost of alternative syphilis intervention scale-up scenarios.

**Methods:**

SITE is a dynamical model of syphilis transmission among adults 15–49 years. Individuals are divided into nine groups based on sexual behaviour and into six stages of infection. The model was calibrated to PNG using data from routine surveillance, bio-behavioural surveys, research studies and program records. Inputs included syphilis prevalence, risk behaviours, intervention coverage and service delivery unit costs. Scenarios compared different interventions (clinical treatment, contact tracing, syphilis screening, and condom promotion) for incidence and cost per infection averted over 2021–2030.

**Results:**

Increasing treatment coverage of symptomatic primary/secondary-stage syphilis cases from 25–35% in 2020 to 60% from 2023 onwards reduced estimated incidence over 2021–2030 by 55%, compared to a scenario assuming constant coverage at 2019–2020 levels. The introduction of contact tracing in 2020, assuming 0.4 contacts per symptomatic person treated, reduced incidence over 2021–2030 by 10%. Increasing screening coverage by 20–30 percentage points from the 2019–2020 level reduced incidence over 2021–2030 by 3–16% depending on the target population. Scaling-up clinical, symptom-driven treatment and contact tracing had the lowest cost per infection averted, followed by condom promotion and periodic screening of female sex workers and men who have sex with men.

**Conclusions:**

PNG could considerably reduce its syphilis burden by scaling-up clinical treatment and contact tracing alongside targeted behavioural risk reduction interventions. SITE is a useful tool countries can apply to inform national STI programming and resource allocation.

## Introduction

Papua New Guinea (PNG) is the largest Pacific island nation and is estimated to have among the highest rates of curable sexually transmitted infections (STI) globally and the highest burden of HIV within the Pacific region (Korenromp et al., 2019; Nishijima et al., 2020). PNG’s public health response to syphilis and other STIs is guided by joint HIV and STIs strategies which outline national targets for reducing prevalence and priority populations for intervention (National AIDS Council of Papua New Guinea, 2010; National Department of Health Papua New Guinea, 2017).

Sexual health programs in PNG are implemented by provincial disease control units that rely on weak vertical over-stretched health systems (National AIDS Council Papua New GuineaAustralian Government Department of Foreign Aid and TradeUNAIDS, 2018). Without the financial and human resources needed to fully implement the strategy, national targets for STI control have so far not been achieved. Among pregnant women accessing antenal care (ANC), syphilis testing and treatement are hampered by regular stock-outs of rapid diagnostic tests and treatment.

Recognizing the severe adverse health outcomes associated with untreated syphilis, and committed to improving health outcomes among priority populations including mothers and babies, the government of PNG is examing new approaches to actualize the national STI control strategy. This includes revising the national HIV testing algorithm to include dual HIV/syphilis tests, a novel community-led HIV and STI testing and case management program, and refocusing condoms distribution.

The Syphilis Interventions Towards Elimination (SITE) model was developed in 2019/2020 as a decision support tool for national HIV/STI programs to explore the impact and cost-per-infection averted of alternative syphilis prevention, screening and treatment interventions or combinations of interventions (Korenromp et al., 2020).

In July 2020, the model was piloted in PNG in collaboration with the National HIV/STIs program, the World Health Organization (WHO) country office and Western Pacific regional office, and Avenir Health. An online workshop was held to review national data inputs, discuss relevant program senarios and calibrate the model to PNG. This paper presents alternative program scale-up scenarios projections developed for PNG, with results over 2021–2030.

## Methods

### Model: structure and population group sizes

SITE is a dynamic, compartmental model that simulates adult syphilis transmission (Korenromp et al., 2020). Adults (15–49 years) are split into seven groups of transmission risk, based on sexual behaviours. In addition, there are groups of women and men who have not yet started sexual activity.

The model was programmed in C++ as an add-on package of R, version 3.5.1 (R Core Team, 2018), and designed for use by national HIV/STI program staff after a short (several-days, workshop-like) training. User-defined parameter input values are specified in an Excel file, as are model outputs. It runs over 1970–2050, in weekly time steps. Software, detailed methods explanation and instructions for users are available at https://avenirhealth.org/software-site.php.

### Natural history

The SITE model distinguishes six infection stages. Susceptible individuals acquire infection at a rate depending on the probability of transmission per sexual contact, number of sexual partners, acts per partner, condom use and the likelihood of encountering an infectious partner. If untreated, an infected individual moves sequentially from incubation stage to primary/secondary syphilis to latent syphilis. Primary and secondary syphilis are combined, as tests used for screening syphilis do not distinguish between these stages and data on differences in infectivity between the two are limited (Garnett et al., 1997; Holmes, 2008; Johnson & Geffen, 2016).

Rates of movement between stages reflect the assumed stage duration for untreated infection and coverage of treatment and screening in each stage. Treatment is assumed to be symptom-driven or screening-based. In addition, individuals in the latent stage can be inadvertently cured when treated for another infection with an antibiotic effective against syphilis. Ultimately, all treated individuals become susceptible to reinfection at the same rates as those who were never infected.

In the model only individuals with primary/secondary infection are infectious. Transmission is modelled via probabilities per sexual act fitted within plausible ranges to reproduce country prevalence data (Supplementary File 1).

### Interventions

The model was used to simulate four types of interventions. Across all interventions, syphilis treatment is assumed to be equally effective (default value 90%) for all population groups (Johnson & Geffen, 2016; Long et al., 2006).

• **Screening followed by treatment for confirmed positives**, ie. positive on both treponemal and non-treponemal Rapid Plasma Reagin (RPR) tests: The effect of treatment is assumed to depend on the infection stage when treated:
  o Primary/Secondary infection: 40% become RPR-negative and are immediately susceptible; the others remain RPR-positive and immune to reinfection;
  o Latent stage: 100% remain RPR-positive and immune to reinfection;
  o Recovered but not yet susceptible (Compartments 4 and 5): 100% are diagnosed and treated but remain in the same stage of infection (Holmes, 2008; Johnson & Geffen, 2016).
• **Clinical treatment of individuals with symptomatic infection:** Only individuals with primary/secondary infection present with symptomatic infection; the probability of being symptomatic is 60%. If treated, 40% become RPR-negative and immediately susceptible to reinfection (Johnson & Geffen, 2016); the rest recover and become non-infectious, but are temporarily RPR-positive and immune to reinfection.
• **Contact referral, testing and treatment, of partners of clinically treated patients.** Tracing results in treatment if the contact is diagnosed with primary/secondary or latent stage infection. Contacts come proportionally from all of the population groups, except Female Sex Workers (FSW) and their clients who are assumed not to refer each other. Prevalence among contacts is calculated dynamically, assuming most contacts tested will typically have been infected by the index case (full mathematical details in (Korenromp et al., 2020)). Modelled prevalence rates of active syphilis among contacts traced (also called test positivity rate or yield) for PNG, ranging from 6 to 56% across the 7 risk groups modelled, for an overall rate of 31% in 2020, were in line with data published contact tracing studies around the world, where prevalence of active syphilis among contacts ranged between 8.8 and 57% (Annex 2 of (Korenromp et al., 2020)).
• **Condom usage:** Modelled as an 80% reduction in the probability of transmission per sexual act, randomly distributed within a population group (Korenromp et al., 2020).

### PNG model parameters

Risk group sizes and behavioural parameter values, including condom use, were fitted using point estimates from Integrated Bio-Behavioural Surveys (IBBS), published literature program data, and values for parameters in the Spectrum-Goals model calibrated to PNG’s HIV epidemic (Table 1a) (Stover et al., 2016).

**Table 1:** Parameter values of the SITE model calibrated to Papua New Guinea.

**a. Population sub-group sizes and their numbers of partners**					
Group (15–49 years)	Share of Population	Married/with stable heterosexual partner	Partners/year	Sex acts/partner/year	Source
Not yet sexually active Women	14%	NA	0	NA	Demographic and Health Surveys (DHS) 2006 (National Statistics Office of Papua New Guinea, 2009) and 2016–2018 (National Statistics Office of Papua New Guinea, 2019)
Low-Risk Women	51%	100%	1	100	DHS, community surveys (Maibani-Michie et al., 2011; National AIDS Council Secretariat, 2012), studies in sexual health clinics (Vallely et al., 2014) and model’s balancing of stable & casual partnerships between women and men
Medium-Risk Women	33%	30%	3 casual	30	
High-Risk Women/FSW	2.3%	12%	55 clients	5	National size estimations (Hakim et al., 2019; Kelly-Hanku et al., 2018; Weikum et al., 2019); IBBS (Hakim et al., 2019; Kelly-Hanku et al., 2018, 2020; Millan et al., 2007); studies (Family Health InternationalUSAID AsiaPapua New Guinea National AIDS Council, 2011; Maibani-Michie & Yeka, 2005; Maibani-Michie et al., 2007; Morineau, 2011; National AIDS Council Secretariat, 2012; Papua New Guinea National AIDS Council SecretariatPartners, 2008; Yeka et al., 2006), 2018 national HIV investment case (National AIDS Council Papua New GuineaAustralian Government Department of Foreign Aid and TradeUNAIDS, 2018)
**All Women**	**100%**				2019 World Population Prospects, 2015–2030 medium variant projections (United Nations Population Division, 2019)
Not yet sexually active Men	14%	NA	0	NA	DHS (National Statistics Office of Papua New Guinea, 2009; National Statistics Office of Papua New Guinea, 2019)
Low-Risk Men	40%	100%	1	100	Fitted, balancing behaviours reported in DHS (National Statistics Office of Papua New Guinea, 2009; National Statistics Office of Papua New Guinea, 2019) community surveys (Buchanan et al., 2010, 2011; Maibani-Michie et al., 2011), studies in sexual health clinics (Vallely et al., 2014) and balancing with FSW/client group size and numbers of FSW/client contacts
Medium-Risk Men	28%	40%	3 casual	30	
High-Risk Men	16%	30%	5 FSW	5	
MSM	1.6%	20% (i.e. bisexual)	5	13	National size estimations (Weikum et al., 2019); IBBS (Hakim et al., 2019; Kelly-Hanku et al., 2018; Millan et al., 2007); studies (Family Health InternationalUSAID AsiaPapua New Guinea National AIDS Council, 2011; Maibani-Michie & Yeka, 2005; Maibani-Michie et al., 2007; Morineau, 2011; National AIDS Council Secretariat, 2012; Papua New Guinea National AIDS Council SecretariatPartners, 2008; Yeka et al., 2006), 2018 national HIV investment case (National AIDS Council Papua New GuineaAustralian Government Department of Foreign Aid and TradeUNAIDS, 2018)
**All men**	**100%**				2019 World Population Prospects, 2015–2030 medium variant projections (United Nations Population Division, 2019)

b. Syphilis intervention coverages historically, currently and as targeted in alternative program scale-up scenarios						
Population group	Historic	Current & Constant-coverage, 2020+	Single-intervention scale-up, 2023+	Scale-up: Moderate	Scale-up: Maximum	Source or estimation method
	1985			2023+ all interventions combined		
**Clinical treatment of symptomatic Primary/Secondary episodes**						
Low- & Medium-risk Women	25%	25%	55%, 60% & 60% (as 1 scenario)	35%	40%	WHO global STI estimations assume 35% for countries with low treatment access; (Newman et al., 2015; Rowley et al., 2019) Authors consider PNG to be among the very lowest-treatment access countries, evidenced by low self-reported use by key populations to any HIV/STI prevention and treatment services (Family Health InternationalUSAID AsiaPapua New Guinea National AIDS Council, 2011; Hakim et al., 2019; Kelly-Hanku et al., 2018; Millan et al., 2007)
High-risk women = FSW	35%	35%		45%	50%	
Men, all including MSM	35%	35%		45%	50%	
**Contact tracing: contacts traced per symptomatic index case treated**						
All groups	0		0.40	0.20	0.40	Program and study data from other countries (Korenromp et al., 2020)
**Screening coverage, per year**						
Low- & Medium-risk Women	0%	10%	30%	20%	25%	Program data (Fig. 1d), HIV/STI and ANC program statistics over 2016–2019 (National Department of Health Papua New Guinea, 2017; National Department of Health Papua New Guinea, 2019)
High-risk women = FSW	10%	18%	48%	28%	33%	MSM & FSW: 2017 national program service reach results (National Department of Health Papua New Guinea, 2019); 90% service coverage for FSW and MSM targeted for 2022 per PNG’s 2018–2022 national HIV/STI strategy (National Department of Health Papua New Guinea, 2017)
Low-risk Men	0%	0%	NA	5%	10%	
Medium-risk Men	0%	4%	NA	9%	14%	
High-risk Men = FSW clients	0%	7%	NA	17%	22%	
MSM	5%	15%	45%	25%	30%	
**Condom use: sex acts protected**						
Medium-risk/casual	1%	15%	35%	25%	30%	IBBS (Hakim et al., 2019; Kelly-Hanku et al., 2018, 2020; Millan et al., 2007); DHS (National Statistics Office of Papua New Guinea, 2009; National Statistics Office of Papua New Guinea, 2019), surveys (Buchanan et al., 2010; Buchanan et al., 2011; Family Health InternationalUSAID AsiaPapua New Guinea National AIDS Council, 2011; Maibani-Michie et al., 2011; Maibani-Michie & Yeka, 2005; Maibani-Michie et al., 2007; Morineau, 2011; National AIDS Council Secretariat, 2012; Papua New Guinea National AIDS Council SecretariatPartners, 2008; Vallely et al., 2014; Yeka et al., 2006), 2018 HIV investment case (National AIDS Council Papua New GuineaAustralian Government Department of Foreign Aid and TradeUNAIDS, 2018). In comparison, PNG’s National HIV strategy (National Department of Health Papua New Guinea, 2017) for 2022 targeted 90% coverage of condom promotion, and 90% condom use in FSW, MSM and medium-risk/casual contacts
High-risk (FSW/client)	1%	53%	83%	63%	68%	
MSM	1%	50%	80%	60%	65%	

Notes to Table 1b: In the single-intervention scale-up scenarios, coverage was assumed to be 30 percentage points higher than at 2019–2020 for all interventions; except for screening and condom promotion for low-risk and medium-risk groups which increased by 20 percentage points as (apart from ANC women) these are not a programmatic target group. The ‘Moderate’ and ‘Maximum’ program packages scaled-up coverage of each of the interventions by 10% or 15% points, respectively, apart from screening and condom promotion for low-risk and medium-risk groups which increased by 5% and 10%.

Historic coverage of syphilis screening was based on annual ANC program data for low- and medium-risk women and IBBS testing and prevention service data for FSW and Men who have sex with Men (MSM). Screening volumes are recorded for pregnant women, but not for other groups. As further input, therefore, modelled volumes of diagnoses resulting from screening (across all groups) combined were compared and adjusted to match program-reported annual diagnoses of individuals positive on both a rapid treponemal and RPR test (called Latent syphilis in PNG).

Coverage of clinical treatment was based on assumptions used by the WHO in global STI estimations for countries with low treatment access (Newman et al., 2015; Rowley et al., 2019). These were verified to be consistent with national case reports of Genital Ulcer Disease (GUD).

The baseline calibration assumed zero coverage of contract tracing from syphilis patients, reflecting negligible numbers recorded by the national program.

### PNG prevalence data

Syphilis prevalence data were compiled to calibrate the model to PNG’s epidemic history (Supplementary File 2). Before fitting, prevalence data were adjusted to reflect diagnostic tests used; studies that did not use both a treponemal and a non-treponemal test were adjusted to a corresponding prevalence of RPR+/TPHA + dual positivity. Test adjustors were based on those used in WHO global and Spectrum-STI country estimates for studies with no RPR titre information (Newman et al., 2015; Nishijima et al., 2020; Rowley et al., 2019). For studies that defined syphilis as TPHA-positive and RPR-positive above a threshold titer of ≥1:8, observed prevalence was increased 2.5-fold. This multiplier was based on studies of dual positivity with and without RPR titer in Bangladesh and Peru (Campos et al., 2006; Haseen et al., 2012; Lama et al., 2010; Sanchez et al., 2007; Tabet et al., 2002). All test-adjusted prevalences were lowered by 10%, the proportion of RPR+/TPHA dually positive samples estimated to be due to yaws (Nishijima et al., 2020). Yaws is endemic in PNG, though somewhat segregated from syphilis by geography (rural versus urban) and ages most affected (children versus adults) (Backhouse et al., 1998; Manning & Ogle, 2002). The maximum adjusted prevalence in any population was limited to 45% in FSW, 35% in MSM, and 20% in lower-risk groups.

### Intervention scenarios and impact assessment

Each intervention was scaled-up individually in one or more population groups (Table 1b). Feasible targets were based on coverage and impact targets of the National HIV/STI Strategy (National Department of Health Papua New Guinea, 2017) in collaboration with workshop participants. Two intervention combination packages (Moderate and Maximum) were also simulated.

All scale-up scenarios assumed coverage increased linearly starting in 2021, reaching target value(s) in 2023 and maintained through 2030. Scale-up scenarios were compared to a constant coverage scenario, where coverage of all interventions remained as in 2019–2020.

### Cost and cost-per-infection-averted

Intervention scenarios were costed applying service delivery unit costs (per test, treatment, condom, or contact traced) to volumes of services. For each intervention the cost-per-infection-averted (in US$) over 2021–2030 was calculated, as an indicator of cost-effectiveness. No discounting was applied to costs or infections.

### Sensitivity analysis

Univariate sensitivity analyses examined the influence of two key uncertainties in the PNG model calibration on incidence reduction and cost-per-infection-averted.

## Results

### SITE calibration to PNG: 1989 to 2020

**Fig. 1:**
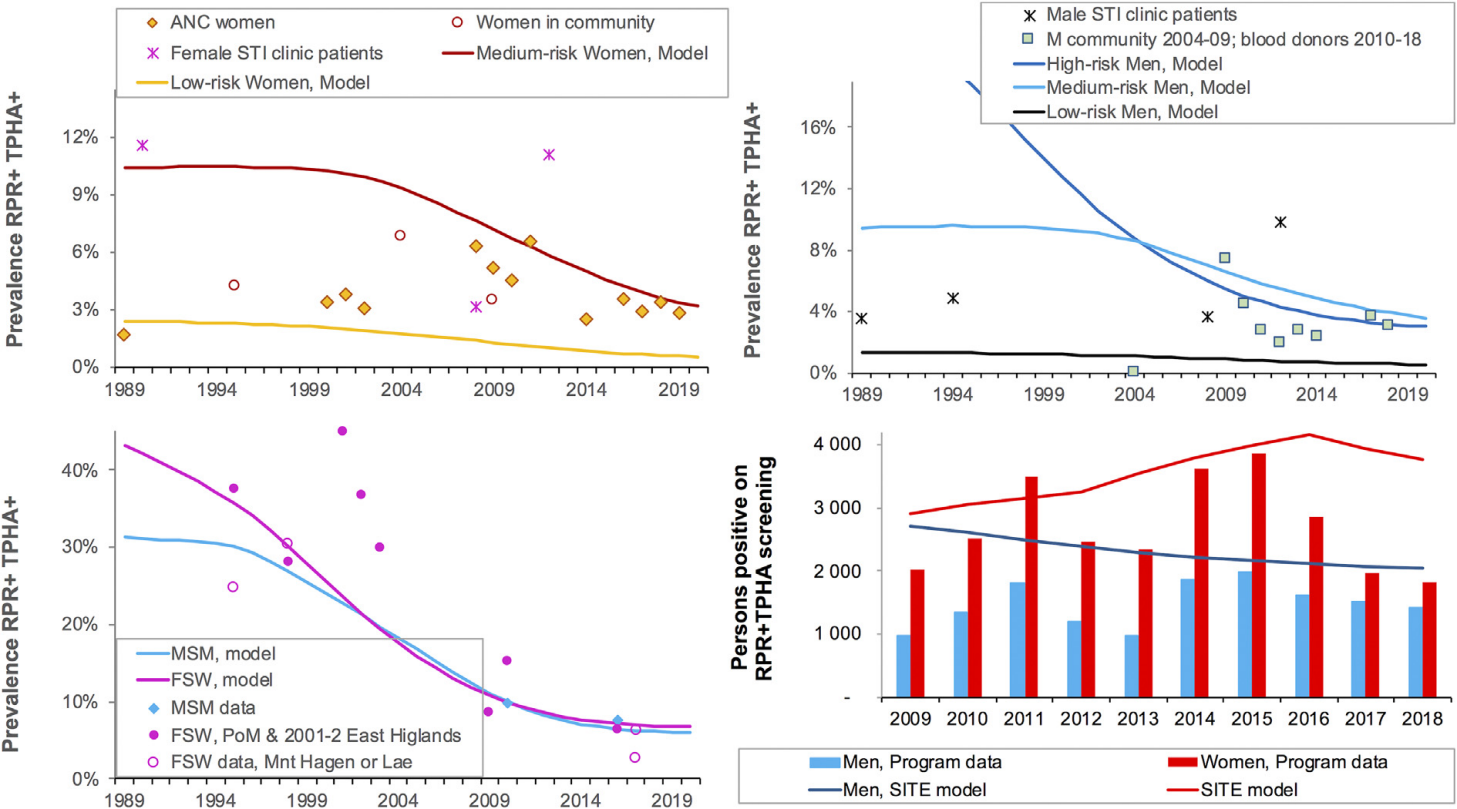
Model fit to syphilis prevalence data and program-reported diagnoses from screening (a) Low- and medium-risk women; (b) Heterosexual men; (c) FSW and MSM; (d) Diagnoses of latent syphilis identified through population and/or clinic-based screening. Notes to Fig. 1. Prevalence data shown after adjustment for diagnostic test performance and endemic yaws (see Methods). In (c), the two data points for FSW in 2017 are from two sentinel cities (Lae, with higher prevalence, and Mount Hagen) (Kelly-Hanku et al., 2020). PoM = Port Moresby, the capital. The MSM 2009 data is from male and transgender sex workers (Kelly-Hanku et al., 2011).

The calibrated PNG model estimated a decline in prevalence in all groups from 1989 to 2020 (Fig. 1a–c). In 2020, highest prevalences were in FSW (6.8%) and MSM (6.1%). FSW and MSM account for only 2.3% of adult women and 1.6% of adult men, but 18.5% and 10% of incident cases respectively, making them important target groups for syphilis interventions. The high incidence in FSW and MSM reflects not only their high exposure, but also their relatively better access to syphilis screening and treatment which renders them susceptible to re-infection. Overall prevalence in 2020 was higher in men than in women, due to large group of high-risk men i.e. FSW clients, with high prevalence (Supplementary File 3).

### Prevalence

Fig. 1a records prevalence data thought to be representative of low- and medium-risk women: ANC women, women in community studies, and STI clinic patients. In most countries STI patients are not considered representative of the general population. In PNG, however, the ‘White house clinics’ are the only clinics providing comprehensive health care in many provinces; therefore data from these clinics were viewed as representative of medium- and high-risk populations. Fig. 1b shows the corresponding data for men, from community studies, STI clinic patients and blood donors (voluntary and family members (Mamu & Varpit, 2016)). Blood donor data were assumed to be representative of heterosexual men (low-, medium- and high-risk). Syphilis screening data are not disaggregated by sex in PNG but 85–87% of blood samples tested for HIV were from men (National Department of Health Papua New Guinea, 2019).

Fig. 1c shows data for FSW and MSM from IBBS studies and other surveys. The decline in prevalence of syphilis in FSW parallels the documented decline in HIV prevalence, precipitated by increased condom use starting around 1995 (National Department of Health Papua New Guinea, 2019; Stover et al., 2016). For MSM only two surveys were identified (2009–10 and 2016–17); whilst suggesting a decline, these do not provide conclusive evidence.

### Treatment volumes and diagnoses from screening

Annual screening-based diagnoses reported by the national program are shown in Fig. 1d. The number of reported diagnoses in women is greater than in men, reflecting the large number of pregnant women screened. SITE results are comparable to these data.

PNG practices syndromic case management for STIs; there are no data on numbers of people treated for syphilis. The national health information system records cases of GUD: in 2018 there were 5616 male plus female cases (National Department of Health Papua New Guinea, 2019). Syphilis is estimated to account for 20–44% of GUD cases – suggesting there had been between 1123 and 2471 treated syphilis cases in 2018. The modelled 1465 people (614 women and 851 men) treated for primary/secondary syphilis in 2018 is within this range (Supplementary File 4).

### Intervention projections: 2021–2030

#### Impact

**Fig. 2:**
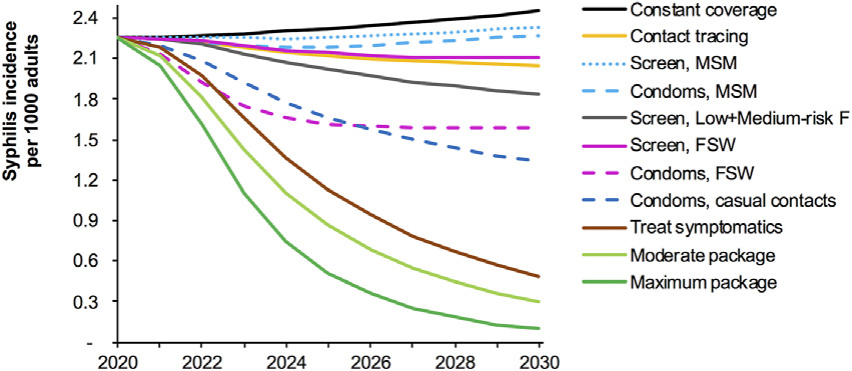
Syphilis incidence rates under alternative prevention, screening and treatment scenarios, Papua New Guinea, 2020–2030.

In the constant-coverage scenario, the annual incidence rate increased slightly throughout 2030 (Fig. 2), reflecting the recent increase in screening coverage, which made more individuals (especially FSW and MSM) available for reinfection. Among the individual interventions, clinical treatment of symptomatic primary/secondary cases had greatest impact on incidence (Fig. 2): over 2021–2030 incident cases fell by 50% compared to cases under Constant-coverage (Table 2). Introduction of Contact tracing reduced incidence by 10% over 2021–2030.

**Table 2:** Service volumes, cost (in US$), infectious averted and cost per infection averted of syphilis control scenarios, 2021–2030, Papua New Guinea.

Intervention Scenario	Persons screened	Contacts traced	Index patient treated	Treatment after screening	Contacts diagnosed	Condoms used	Cost, 2021–2030, including condoms:		Infections averted, 2021–2030, from Constant Coverage		Cost per infection averted, incl. Condoms:	
							at full cost	at 20% shared cost	Number	%	at full cost	at 20% shared cost
Constant coverage	2,756,172		17,764	55,288		203,312,110	26,969,185	10,192,615				
Screening, Low + Medium-risk Women	6,429,685		15,240	77,796		203,312,110	34,623,716	17,847,145	20,964,168	16%	0.37	0.37
Screening, FSW	2,906,932		16,192	57,808		203,312,110	27,280,766	10,504,196	12,063,764	9%	0.03	0.03
Screening, MSM	2,866,565		17,196	56,824		203,312,110	27,199,555	10,422,984	4,280,746	3%	0.05	0.05
Contact tracing	2,756,172	9673	16,071	49,834	3026	203,312,110	26,975,865	10,199,295	13,698,159	10%	0.0005	0.0005
Clinical treatment, symptomatic cases	2,756,172		14,742	42,764		203,312,110	26,905,425	10,128,855	72,410,867	55%	−0.0009	−0.0009
Condoms, FSW/client	2,756,172		12,819	47,392		245,847,458	32,866,410	11,325,881	38,108,090	29%	0.15	0.03
Condoms, MSM	2,756,172		16,814	53,292		210,669,655	27,986,380	10,385,765	7,488,483	6%	0.14	0.03
Condoms, Medium-risk/casual contacts	2,756,172		13,275	49,777		339,407,294	36,449,575	12,051,674	40,896,028	31%	0.23	0.05
Program scale-up package: Moderate	5,840,550	2500	9245	65,383	408	289,444,547	40,527,210	17,996,128	84,166,302	63%	0.16	0.09
Program scale-up package: Maximum	7,681,049	3677	7324	69,904	525	331,072,877	47,832,932	22,504,757	98,468,063	74%	0.21	0.13

Notes to Table 2. Clinical treatment is the one cost-saving intervention, with a negative cost per infection averted. Unit costs assumed for scenario costing: Screening per adult: **US$ 2.1**, based on 2012 bulk procurement data reported to WHO also quoted in a global congenital syphilis investment case analysis (Kahn et al., 2014); 1 adult with confirmed syphilis treated for syphilis (3 doses of benzathine penicillin; labor and supplies & counseling): **US$ 5.8,** based on a global congenital syphilis investment case analysis, quoting 2012 bulk procurement data reported to WHO (Kahn et al., 2014), built up of $ 3.7 for treatment + counselling & $ 2.1 for testing prior to treatment; 1 syphilis-adult contact-traced and treated: **US$ 5.5,** which includes the tracing activity but excludes the cost of testing contacts of index cases, which was costed at $ 2.1 per contact tested, applied to the volume of contacts shown in column ‘Contacts traced’; 1 condom distributed (including procurement, distribution and promotion/counselling): **US$ 0.14** for FSW and MSM, based on the Asia regional estimate in Avenir Health’ global unit cost repository (Avenir Health HIV Unit Cost Repository, 2019) **$0.07** for medium-risk beneficiaries with casual partnerships.

For screening, at modelled coverages, impact was greater when targeting medium- and low-risk women and/or FSW than MSM, reflecting larger numbers reached.

Increasing condom use in either FSWs or MSM was more effective than screening, at modelled coverage levels. For both condom promotion and screening, targeting FSW had a larger population-wide effect than targeting MSM, owing to relative population sizes and the fact that MSM mix primarily among themselves.

The moderate and maximum intervention packages reduced incidence over 2021–2030 by 63% and 74% compared to Constant-coverage, averting 84 and 98 million cases respectively (Table 2).

#### Cost and cost-per-infection-averted

The cost of syphilis prevention, screening and treatment throughout 2021–2030 at 2019–2020 coverage levels was estimated at US$ 27.0 million if the full cost of condoms is included (Table 2). However, if condom costs are shared with other programs (e.g. HIV/AIDS) with syphilis responsible for only 20% then the cost falls to US$ 10.2 million.

Clinical treatment of symptomatic cases had the lowest cost-per-infection-averted among the four interventions and over 2021–2030 was cost-saving, as falling incidence reduced treatment costs. Contact tracing had a negligible cost-per-infection averted (<US$ 0.001), as it identifies and treats infectious patients. Condom usage had the next lowest cost-per-infection-averted, especially if costs are shared. For condom promotion the lowest cost-per-infection-averted was for FSW and MSM contacts (US$ 0.03, with cost sharing), followed by medium-risk contacts (US$ 0.05).

Cost-per-infection-averted of screening was US$ 0.03 for FSW and US$ 0.05 for MSM. Cost-per-infection-averted of screening low-risk and medium-risk women was much higher, reflecting the lower prevalence in these populations. Whilst syphilis screening − if well-targeted − has a low cost-per-infection-averted, its total cost may be prohibitively high. For 2020 PNG’s HIV/STI budget totaled just under US$ 30 million (National AIDS Council Papua New GuineaAustralian Government Department of Foreign Aid and TradeUNAIDS, 2018), and spending in 2019 totaled around US$ 24 million (National Department of Health Papua New Guinea, 2020) – down from substantially higher annual budgets up to 2014 (National Department of Health Papua New Guinea, 2017). Annual screening of FSW and/or MSM would add US$ 30,000 annually for each group; and screening of lower-risk women up to US$ 800,000 annually.

Cost-per-infection-averted for both multi-intervention packages was within the range of the individual interventions. This was more favorable for the moderate than the maximum package (US$ 0.09 and US$ 0.13, with condom cost shared), due to larger saturation causing diminishing marginal returns once prevalence and incidence fall, under the maximum package.

### Sensitivity analyses

The first analysis varied the proportion of MSM/bisexual men with a stable female partner. Halving this proportion lowered the reduction in incident cases and increased cost-per-infection-averted over 2021–2030 achieved by MSM screening and condom promotion targeted to MSM by 8–10%. Conversely, doubling the MSM marriage rate improved impact and cost-per-infection-averted of these interventions by 16–19% (Supplementary File 5). Relative and absolute impact and cost-per-infection-averted for non-MSM-targeted interventions did not materially change.

In the second analysis, the historic calibration was adjusted to reflect uncertainty in FSW prevalence data: prevalence in FSWs in 2019–2020 was 39% lowered from the default and incidence 46% lower. This had a particular impact on FSW-targeted interventions. The number of incident cases averted over 2021 to 2030 was 19% less than in the default projection for FSW screening and 10% less for FSW condom promotion, with corresponding worsening of cost-per-infection averted for these FSW interventions. In this alternative calibration, medium-risk groups and MSM account for a larger share of incident cases and the reduction in incidence and cost-per-infection-averted of targeting these groups were more favorable (Supplementary File 5).

## Discussion

This was the first country application of SITE, a model to explore the impact of different syphilis interventions on syphilis incidence and cost-per-infection averted. Calibrated to PNG’s national syphilis epidemic and program data, SITE is being used by PNG’s HIV/STI program to identify priority syphilis interventions and reasonable coverage and impact targets for next plans and strategies.

Model results confirmed the importance and favorable cost-per-infection in PNG of STI screening and condom promotion for FSW and MSM, although the sensitivity analyses illustrated uncertainties in their quantitative ranking, reflecting current FSW prevalence and, for MSM/bisexual men, their extent of mixing with the heterosexual population. For FSW and MSM, annual screening and condom promotion had similar cost-per-infection-averted. The roll-out of Pre-Exposure Prophylaxis for HIV offers new opportunities for expanding STI screening at a low cost to individuals who identify as being at high risk.

PNG’s high STI rates and limited laboratory capacity mean syndromic case management policy is a sensible choice for the time being. However − whether syndromic or etiology (test-based) − clinic-based treatment does not reach people with asymptomatic infection, or those who do not seek treatment. The simulations show that introducing contact tracing of etiologically confirmed syphilis cases would be a useful complement to clinical treatment. Contact tracing, by identifying early-stage infectious syphilis cases, helps suppress transmission and reduce future treatment needs, and over the 2021–2030 horizon incurs a negligible cost-per-infection averted.

The model was calibrated using prevalence data from PNG. These data, however, are disproportionately from urban settings and Highland provinces. As a result, the calibrated model may not accurately reflect levels and trends nation-wide. PNG’s urban and highland areas historically had the highest rates of HIV, STI and sexual risk behaviours (National Statistics Office of Papua New Guinea, 2019) and as a result have been prioritized by HIV/STI programs and hence declines in syphilis prevalence and risk behaviours in these areas may be greater than in the rest of the country. Periodic monitoring of STI prevalence and STI-related service usage in rural populations, notably FSW and MSM but also heterosexual men and non-pregnant women, would help to refine and improve future program scenario modelling and program planning. Besides population surveys, clinical surveillance of the etiologies of STI syndromes such as GUD could inform treatment policies.

### Limitations

The validity of modelling results reflect the model’s structure and uncertainties in inputs. In SITE, each of seven modelled sexually active populations is assumed to be homogeneous, with STI exposure and access to screening and treatment distributed equally. In reality, each population has gradients of risk and service uptake, and some individuals may not be reachable or identify as part of the group. More heterogeneity complicates elimination (Mitchell et al., 2013) and SITE may be optimistic regarding the impact of interventions targeting specific populations such as FSW and MSM. Other structural limiations of the current version of the model are discussed in more detail in (Korenromp et al., 2020).

SITE was calibrated on prevalence data, but underlying incidence remains uncertain. For a given prevalence, underlying incidence varies with rates of treatment (clinic-based and following screening) and of incidental cure from latent syphilis, for which population-based data were limited in PNG. This adds uncertainty to the absolute numbers of infections averted by any of the intervention scenarios presented.

The different interventions were compared in terms of their impact on syphilis incidence and cost-per-infection-averted in adults. This is a narrow perspective which ignores important other benefits, e.g. ANC-based syphilis screening improving new-born health, or condom promotion for family planning and preventing other STIs. In addition, country-specific unit costs of intervention service delivery were lacking; instead we used global unit costs. Despite these uncertainties, we believe the patterns of impact and cost-per-infection-averted across interventions and target groups are robust.

## Conclusions

In resource-poor settings prioritizing interventions is vital as countries strive to eliminate syphilis. The SITE model is a new tool that national HIV/STI programs can use to inform and improve strategic STI control planning and interventions optimization. The presented pilot application in PNG showed that PNG has considerable scope to reduce its syphilis burden by scaling-up clinical treatment and contact tracing, alongside behavioural risk reduction interventions and well-targeted screening services already implemented by the HIV/STI control program.

## Funding

Financial support for this work was provided by the World Health Organization (WHO) PNG Country Office.

## CRediT authorship contribution statement

**Shepherd Machekera:** Data curation, Formal analysis, Writing – original draft, preparation. **Peniel Boas:** Data curation. **Poruan Temu:** Data curation. **Zimmbodilion Mosende:** Data curation, Formal analysis. **Namarola Lote:** Data curation. **Angela Kelly-Hanku:** Data curation. **S. Guy Mahiane:** Software, Methodology, Writing – original draft, preparation. **Robert Glaubius:** Software, Methodology, Validation, Writing – review & editing. **Jane Rowley:** Writing – review & editing, Validation, Visualization. **Anup Gurung:** Conceptualization, Funding acquisition, Project administration, Writing – review & editing, Supervision. **Eline Korenromp:** Conceptualization, Formal analysis, Investigation, Writing – original draft, preparation, Supervision.

## Declaration of competing interest

The authors declare that they have no known competing financial interests or personal relationships that could have appeared to influence the work reported in this paper.
