# Supplementary material for: Strategic options for syphilis control in Papua New Guinea– impact and cost-effectiveness projections using the syphilis interventions towards elimination (SITE) model
Source: Infect Dis Model. 2021 Mar 20;6:584–97. doi: 10.1016/j.idm.2021.03.004 (PMC8039768; doi:10.1016/j.idm.2021.03.004)
Supplement: Multimedia component 2 [file mmc2.docx]

**Supplementary Files to :**

**Strategic options for syphilis control in Papua New Guinea– impact and cost-effectiveness projections
using the *Syphilis Interventions towards Elimination (SITE)* model**

*Version 21 October 2020*

**Supplementary File 1.** **Biomedical parameters, and their values in the SITE model’s calibration to Papua New Guinea**

| **Parameter** (compartment of natural history flow chart) | **Value & unit** | **Uncertainty range** | **Source & comments** |
| --- | --- | --- | --- |
| ***Duration of infection:*** | | | South Africa model [1] and WHO global estimates [2-4] |
| Incubation (1) | 4 weeks | 2-6 |  |
| Primary + secondary stage, untreated (2) | 28 weeks | 18-78 | SITE does not explicitly distinguish symptomatic, infectious recurrences during the latent stage; in compensation, we have set the duration of the initial infectious phase as slightly longer than the commonly assumed (or quoted) 18-26 weeks [1, 5-10] |
| Latent (3) untreated, but considering an average rate of incidental cure, from antibiotic exposure to other infections | 780 weeks | 260-1,560 | We assumed an average duration of 15 years, as in WHO global STI estimates [2-4]. This value considers that a substantial proportion of latent cases gets shortened by incidental cure from exposure to oral penicillin, tetracyclines like doxycyclin, macrolides like azithromycin or other antibiotics taken for skin, respiratory and other non-STI-infections [11, 12] |
| Recovered after treatment (4) | 26 weeks | 3-260 | [1] |
| Incidentally cured, after latent stage (5) | 130 weeks | 3-260 | [1] |
| Susceptible to reinfection (6) | Dynamic result | | [1] Dynamic calculation of the Force of infection, see [13] |
| ***Treatment of Primary/secondary cases:*** | | |  |
| Primary/Secondary cases that are symptomatic | 60% | 40-85% | [1-4] |
| Probability of cure when treated, after clinic attendance for symptoms of Primary/Secondary infection | 90% | 61%-95% | The 90% [1] is conservatively low, to account for the lower effectiveness of single-dose treatments against latent syphilis, and people being re-infected by their (stable) partners, absent contact referral |
| Primary + Secondary stage cases who upon treatment turn RPR-seronegative immediately | 40% |  | [1] |
| ***Transmission probabilities per sexual act:*** | | |  |
| Woman-to-man | 0.039 | 0.0005-0.20 | [1, 14-16]. Only individuals in the Primary + Secondary stage are infectious. |
| Man-to-woman | 0.079 | 0.0008-0.30 | [1, 14-16], applied during Primary + Secondary stage only |
| MSM | 0.09 | 0.001-0.10 | [7-9, 16, 17], applied during Primary + Secondary stage only |
| Reduction in transmission probability, per act, from condom usage | 80% | 80-90% | [16, 18-22]. The model distributes condom usage randomly over all relationships and contacts (within each combination/pair of risk groups). In reality, usage clusters in a subset of relationships and is hence more effective; however higher condom efficacy, when combined with survey-based usage rates, would result in historic syphilis declines larger than suggested by syphilis surveillance data |

**Supplementary File 3. Modelled distribution of populations and prevalent and incident syphilis infections, adults 15-49 years, Papua New Guinea, 2020**

 **Supplementary File 4. Syphilis and Genital Ulcer Disease case reporting, and studies of the etiology of Genital Ulcer Disease in PNG**

PNG implements syndromic STI case management. Cases of ulcerative STIs are recorded as Genital Ulcer Disease (GUD) and annual data by gender and province (22 provinces) are available from the national health information system.

Table S4A shows the number of reported GUD cases for 2009-2018 and the SITE-modelled volumes of number of symptomatic Primary/Secondary syphilis cases treated.

The etiology of GUD etiology is not part of routine surveillance in PNG. A GUD etiology study in STI clinics in 5 towns in PNG in 1989-1990 found 20% and 44% of male and female GUD cases to be due to syphilis (Table S4B).[23] This proportion is consistent with the share of syphilis among clinical GUD cases in other countries at that time, although some countries have since reported a shift toward less syphilis (and less chancroid) relative to HSV-2 as the main etiology.[24, 25]

In the model in 2018, 616 men and 399 women were clinically treated for primary/secondary syphilis (Table S4A). This is 23% and 17% of the male and female GUD cases reported in 2018 (Table S4A), consistent with the program-reported GUD case numbers and PNG’s GUD etiological distribution as reported in 1989-90.

The 2018 model estimates of women and men diagnosed with active syphilis during screening, however, are slightly greater than the program-reported diagnoses of ‘latent’ syphilis cases (Table S4A).

**Table S4A. Clinical diagnoses and diagnoses from syphilis screening, in PNG national program data and the SITE model calibration**

|  | **NHIS/Program** | | **SITE model** | | **SITE model** | | **NHIS/Program** | | | **SITE model** | |
| --- | --- | --- | --- | --- | --- | --- | --- | --- | --- | --- | --- |
|  | **Genital Ulcer Disease** | | **Primary/Secondary syphilis cases clinically treated** | | **Clinical syphilis treatments, as % of program-recorded GUD cases** | | **Latent syphilis = RPR+ TPHA+ on screening** | | | **RPR+ TPHA+  on screening** | |
| **Year** | **Men** | **Women** | **Men** | **Women** | **Men** | **Women** | **Men** | **Women incl. ANC** | **ANC only** | **Men** | **Women** |
| 2009 | 5,363 | 5,290 | 868 | 662 | 16% | 13% | 976 | 2,025 |  | 2,722 | 2,918 |
| 2010 | 3,330 | 3,254 | 843 | 642 | 25% | 20% | 1,337 | 2,507 |  | 2,607 | 3,047 |
| 2011 | 3,079 | 2,749 | 826 | 627 | 27% | 23% | 1,819 | 3,492 |  | 2,488 | 3,156 |
| 2012 | 2,427 | 2,264 | 817 | 617 | 34% | 27% | 1,207 | 2,463 |  | 2,383 | 3,248 |
| 2013 | 2,181 | 1,981 | 813 | 610 | 37% | 31% | 966 | 2,333 |  | 2,293 | 3,536 |
| 2014 | 3,002 | 2,216 | 814 | 606 | 27% | 27% | 1,855 | 3,618 |  | 2,217 | 3,783 |
| 2015 | 2,886 | 2,380 | 819 | 604 | 28% | 25% | 1,995 | 3,879 |  | 2,156 | 3,989 |
| 2016 | 2,785 | 2,030 | 826 | 605 | 30% | 30% | 1,622 | 2,867 | 1,310 | 2,108 | 4,159 |
| 2017 | 2,608 | 2,217 | 836 | 608 | 32% | 27% | 1,510 | 1,981 | 1,724 | 2,072 | 3,949 |
| 2018 | 2,978 | 2,638 | 851 | 614 | 29% | 23% | 1,409 | 1,816 | 867 | 2,048 | 3,773 |
| 2019 |  |  | 869 | 623 |  |  |  |  | 1,095 | 2,036 | 3,630 |

Note to Table S4A. Sources of program data: National Health Information System (NHIS), PNG HIV/AIDS annual surveillance and program reports.[26-30]

**Table S4B. Genital Ulcer Disease etiology in a study in Papua New Guinea**

| **Sites** |  | **Port Moresby, Lae, Goroka, Rabaul & Daru** | |
| --- | --- | --- | --- |
| **Years** |  | 1989-90 | |
| **Population** |  | Women | Men |
| **Diagnosis method** | Overall | Clinical | |
|  | HSV-2 | Serology | |
|  | Syphilis | VDRL (≥1:4) + TPHA | |
|  | Chancroid | Culture for H. ducreyi | |
|  | Donovanosis | ? |  |
| **Etiological & clinical diagnosis** | HSV-2 / herpes genitalis | 4 | 23 |
|  | Syphilis | 7 | 9 |
|  | Chancroid | NA | NA |
|  | Donovanosis | 5 | 14 |
|  | } All GUD | **16** | **46** |
|  | Syphilis as % of GUD | **44%** | **20%** |
| **Syphilis serology, all STI patients** | Positive | 9 | 10 |
|  | Syphilis seroprevalence | 12% | 5% |
|  | **Sero-tested** | **78** | **203** |
| **Source** |  | [23] | |

Notes to Table S4B. GUD = Genital Ulcer Disease; HSV-2 = Herpes Simplex Virus type 2; TPHA = Treponema Pallidum Hemagglutination Assay; VDRL = Venereal Disease Research Laboratory test i.e. a non-treponemal test to diagnose syphilis, similar to the RPR.

**Supplementary File 5. Detailed results of sensitivity analyses**

**Sensitivity analysis 1: Proportion of MSM who are married i.e. in a stable heterosexual relationship**

In this analysis, the proportion of MSM/bisexual men with a stable female partner was varied. In the default scenario it was assumed that 20% of MSM had a stable female partner i.e. were married. In the sensitivity analysis, this was halved and doubled.

**Figure S5a. Prevalence calibration for MSM with varying % of MSM married i.e. in stable heterosexual relationship**

**Figure S5b. Incidence rates 2020-2030, by intervention scenario (lines) and by calibration/sensitivity analysis variant (panels)**

Notes to Figure S5b. The middle panel is the same as Figure 2 in the main Results section; copied here for reference.
PoM = Port Moresby, the capital city.

**Table S5a. Reduction in incident cases (all men and women nation-wide) and cost per infection averted, over 2021-2030 relative to Constant-coverage scenario, by intervention scenario (rows) and by calibration/sensitivity analysis variant (columns)**

Notes to Table S5a. Cost-per-infection averted (in US $) adopted the 20% cost share for condoms.

The ‘20% (default)’ columns repeat the results of Table 2 in the main Results section.

**Sensitivity analysis 2: Lowered prevalence and incidence of FSW in 2017, the year with last data**

In this analysis the model was recalibrated to take into account a 2017 data point with lower prevalence than 2 other 2016-2017 data points, and believed to probably be les representative. Specifically, this calibration fitted the mid-point of the 2016 Port Moresby and 2017 Lae and Mount Hagen data points. These three (largest) cities of PNG were sites in intervention/study areas with possibly better treatment and prevention access, and hence lower prevalence than the overall national FSW population.[31]

The lowering of FSW rates was accomplished by reducing the number of clients per FSW per year over 2009-2020, while proportionally increasing the number of FSWs in proportion to the year-specific client frequency reduction, such that overall population syphilis rates were similar to the default calibration.

**Figure S5c. Prevalence calibration for FSW:** (left) default calibration, fitting FSW prevalence to the IBBS data from Port Moresby (PoM) in 2016 and Lae in 2017 (as in Figure 1c in main Results); (right) alternative calibration fitting to Port Moresby 2016, Lae 2017 and Mount Hagen 2017.

**Figure S5d. Incidence rates 2020-2030, by intervention scenario (lines) and by calibration/sensitivity analysis variant (panels)**

Note to Figure S5d. The left-hand panel is the same as Figure 2 in the main Results section; copied here for reference.

**Table S5b. Reduction in incident cases (all men and women, nation-wide) and cost per infection averted, over 2021-2030 relative to Constant-coverage scenario, by intervention scenario (rows) and by calibration/sensitivity analysis variant (columns)**

Notes to Table S5b. Cost-per-infection averted (in US $) adopted the 20% cost share for condoms.

The ‘Default’ columns repeat the results of Table 2 in the main Results section.

**References for Supplementary Files 1, 3, 4 and 5:**

1. Johnson, L. F., Geffen, N. (2016). A Comparison of Two Mathematical Modeling Frameworks for Evaluating Sexually Transmitted Infection Epidemiology. *Sex Transm Dis*. *43*: 139-46. 10.1097/OLQ.0000000000000412.

2. World Health Organization (2011). Prevalence and incidence of selected sexually transmitted infections -- Chlamydia trachomatis, Neisseria gonorrheae, syphilis and Trichomonas vaginalis. Methods and results used by WHO to generate 2005 estimates. Geneva. <http://apps.who.int/iris/bitstream/10665/44735/1/9789241502450_eng.pdf>

3. Newman, L., Rowley, J., VanderHoorn, S., et al. (2015). Global estimates of the prevalence and incidence of four curable sexually transmitted infections in 2012 based on systematic review and global reporting. *PLoS One*. *10*: e0143304. 10.1371/journal.pone.0143304.

4. Rowley, J. T. F., Van der Hoorn, S., Korenromp, E., et al. (2019). Chlamydia, gonorrhoea, trichomoniasis and syphilis: global prevalence and incidence estimates, 2016. *Bull WHO*. *97*: 548-62P. 10.2471/BLT.18.228486.

5. Holmes, K. (2008). *Sexually Transmitted Diseases.* 4th ed. New York City: McGraw-Hill Medical.

6. Garnett, G. P., Aral, S. O., Hoyle, D. V., et al. (1997). The natural history of syphilis. Implications for the transmission dynamics and control of infection. *Sex Transm Dis*. *24*: 185-200.

7. Tuite, A. R., Shaw, S., Reimer, J. N., et al. (2018). Can enhanced screening of men with a history of prior syphilis infection stem the epidemic in men who have sex with men? A mathematical modelling study. *Sex Transm Infect*. *94*: 105-10. 10.1136/sextrans-2017-053201.

8. Tuite, A. R., Fisman, D. N., Mishra, S. (2013). Screen more or screen more often? Using mathematical models to inform syphilis control strategies. *BMC Public Health*. *13*: 606. 10.1186/1471-2458-13-606.

9. Saad-Roy, C. M., Shuai, Z., Van den Driessche, P. (2016). A mathematical model of syphilis transmission in an MSM population. *Mathematical Biosciences*. *277*: 59-70.

10. Pourbohloul, B., Rekart, M. L., Brunham, R. C. (2003). Impact of mass treatment on syphilis transmission: a mathematical modeling approach. *Sex Transm Dis*. *30*: 297-305.

11. South Africa Ministry of Health (2000). National HIV and Syphilis Sero-Prevalence Survey of women attending Public Antenatal Clinics in South Africa 2000. Johannesburg.

12. Bolan, R., Amezola, P., Kerndt, P. J., et al. (2005). Inadvertent Use of Bicillin® C-R to Treat Syphilis Infection --- Los Angeles, California, 1999–2004. *MMWR Morb Mortal Wkly Rep*. *54*: 217-19.

13. Korenromp, E. L., Mahiané, G., Glaubius, R., et al. (2020). The Syphilis Interventions towards Elimination (SITE) model projecting epidemic impact and cost of syphilis prevention and treatment interventions – technical methods report. <https://avenirhealth.org/software-site.php> Accessed 03 September 2020.

14. Hontelez, J. A., Lurie, M. N., Barnighausen, T., et al. (2013). Elimination of HIV in South Africa through expanded access to antiretroviral therapy: a model comparison study. *PLoS Med*. *10*: e1001534. 10.1371/journal.pmed.1001534.

15. Korenromp, E. L., Van Vliet, C., Grosskurth, H., et al. (2000). Model-based evaluation of single-round mass STD treatment for HIV control in a rural African population. *AIDS*. *14*: 573-93.

16. Stoltey, J. E., Cohen, S. E. (2015). Syphilis transmission: a review of the current evidence. *Sex Health*. *12*: 103-9. 10.1071/SH14174.

17. Gray, R. T., Hoare, A., Prestage, G. P., et al. (2010). Frequent testing of highly sexually active gay men is required to control syphilis. *Sex Transm Dis*. *37*: 298-305. 10.1097/OLQ.0b013e3181ca3c0a.

18. Holmes, K. K., Levine, R., Weaver, M. (2004). Effectiveness of condoms in preventing sexually transmitted infections. *Bull World Health Organ*. *82*: 454-61.

19. Stover, J., Rosen, J. E., Carvalho, M. N., et al. (2017). The case for investing in the male condom. *PLoS One*. *12*: e0177108. 10.1371/journal.pone.0177108.

20. Koss, C. A., Dunne, E. F., Warner, L. (2009). A systematic review of epidemiologic studies assessing condom use and risk of syphilis. *Sex Transm Dis*. *36*: 401-5. 10.1097/OLQ.0b013e3181a396eb.

21. Fitch, J. T., Stine, C., Hager, W. D., et al. (2002). Condom effectiveness: factors that influence risk reduction. *Sex Transm Dis*. *29*: 811-7.

22. Paz-Bailey, G., Shah, N., Creswell, J., et al. (2012). Risk behaviors and STI prevalence among people with HIV in El Salvador. *Open AIDS J*. *6*: 205-12. 10.2174/1874613601206010205.

23. Hudson, B. J., van der Meijden, W. I., Lupiwa, T., et al. (1994). A survey of sexually transmitted diseases in five STD clinics in Papua New Guinea. *P N G Med J*. *37*: 152-60.

24. Becker, M., Stephen, J., Moses, S., et al. (2010). Etiology and determinants of sexually transmitted infections in Karnataka state, south India. *Sex Transm Dis*. *37*: 159-64. 10.1097/OLQ.0b013e3181bd1007.

25. Rietmeijer, K. C. (2016, May). The aetiology of STI syndromes in Zimbabwe. 17th IUSTI World Congress;Marrakech. Marrakechp. 117.

26. National Department of Health STI/HIV/AIDS Surveillance Unit Papua New Guinea (2012). The 2010 National STI, HIV and AIDS annual Surveillance report, Papua New Guinea. Port Moresby.

27. National Department of Health STI/HIV/AIDS Surveillance Unit Papua New Guinea (2010). The 2009 National STI, HIV and AIDS annual Surveillance report, Papua New Guinea. Port Moresby. <https://www.aidsdatahub.org/sites/default/files/documents/2009_STI_and_HIV_Annual_Surveillance_Report.pdf>

28. National Department of Health STI/HIV/AIDS Surveillance Unit Papua New Guinea (2013). The 2011 National STI, HIV and AIDS annual Surveillance report, Papua New Guinea. Port Moresby.

29. National Department of Health Papua New Guinea (2019). 2018 STI/HIV and AIDS Program Annual Report. Port Moresby.

30. Papua New Guinea National Department of Health: STI HIV/ AIDS Surveillance Unit (2017). The 2012-2016 National STIs, HIV/AIDS Surveillance report, Papua New Guinea. Port Moresby.

31. Kelly-Hanku, A., Weikum, D., Badman, S. G., et al. (2020). Factors associated with HIV and syphilis infection among female sex workers in three cities in Papua New Guinea: findings from Kauntim mi tu, a biobehavioral survey. *Sex Health*. 10.1071/SH19218.
